# Surgical resilience may represent the perioperative expression of dynamic adaptive coping

**DOI:** 10.1097/PR9.0000000000001501

**Published:** 2026-10-13

**Authors:** Cecília Daniele de Azevedo Nobre, Bruno Vítor Martins Santiago

**Affiliations:** a Universidade do Estado do Rio de Janeiro (UERJ), Rio de Janeiro, Brazil; b Universidade Federal do Rio de Janeiro (UFRJ), Rio de Janeiro, Brazil

## Letter to the Editor:

We read with interest the article by Blandhol et al.,^2^ *Rethinking coping in chronic pain: a systematic review and dynamic interaction model of coping*, which reconceptualizes coping as a dynamic process governed by controllability, predictability, salience, resources, and motivation rather than static adaptive or maladaptive strategies. We believe that this model can be extended to perioperative medicine through the proposed Surgical Resilience Framework, an extension tailored to the demands of surgical stress.

Unlike chronic pain, surgery is a predictable, time-limited, biologically measurable, and clinically modifiable stressor. The perioperative period provides a unique adaptive model in which psychological, behavioral, and physiological responses evolve from preoperative anticipation to postoperative recovery. Unlike chronic pain, where adaptation unfolds over prolonged and unpredictable trajectories, surgery offers a temporally bounded model in which dynamic coping, physiological stress regulation, and functional recovery can be observed as an integrated adaptive system. This context provides conceptual insights into adaptive processes and establishes the perioperative setting as a translational model for resilience research.^1,4–6^

We propose the Surgical Resilience Framework as a conceptual extension of the Dynamic Interaction Model. The framework conceptualizes perioperative recovery as the interaction between psychological adaptation and physiological stress regulation, mediated by the model's 5 domains and resulting in individualized recovery trajectories. Accordingly, it generates testable hypotheses linking biological reserve, adaptive coping, and postoperative recovery, providing a basis for empirical validation. We define surgical resilience as the dynamic capacity to preserve or regain physiological, psychological, and functional equilibrium throughout the perioperative continuum.^1,8^ It differs from coping, adaptation, and recovery, representing an integrative adaptive capacity emerging from interactions among biological reserve, psychological flexibility, behavioral engagement, and contextual resources.

Because surgical stress is acute, biologically intense, temporally constrained, and therapeutically modifiable, the 5 domains require perioperative refinement. Resources encompass physiological reserve, frailty, nutritional status, inflammatory capacity, and cardiopulmonary fitness alongside psychosocial assets. Predictability includes procedural planning and expectation management; controllability, perceived self-efficacy and participation in recovery; salience, the biological and psychological significance of acute postoperative pain; and motivation, adherence to mobilization, rehabilitation, and self-management.^1,2,5,8^

These domains interact with biological mechanisms activated by surgical stress. Neuroendocrine activation, systemic inflammation, immune modulation, nociceptive signaling, emotional regulation, and expectation updating jointly shape postoperative pain, central sensitization, functional recovery, opioid requirements, and the transition to persistent postsurgical pain. Perioperative adaptation emerges from reciprocal interactions between psychological coping and physiological stress regulation.^4,5^

Enhanced Recovery After Surgery provides an evidence-based, multimodal framework for standardizing perioperative care and accelerating recovery.^7^ However, standardized pathways do not fully account for interindividual variability in postoperative recovery trajectories. The Surgical Resilience Framework may complement ERAS by providing an adaptive link between standardized perioperative interventions and individualized recovery, supporting resilience assessment, prehabilitation, personalized analgesia, and psychological optimization as steps toward precision perioperative medicine^1,3,4,8^

We congratulate Blandhol et al.^2^ for advancing a dynamic model of coping. We propose that surgical resilience be viewed as the observable perioperative manifestation of dynamic adaptive coping under acute biological stress. This framework may provide a unifying conceptual model bridging pain science, perioperative physiology, and precision perioperative medicine.

## Disclosures

The authors have no conflict of interest to declare.
